# Comparing national dementia plans and strategies in Europe – is there a focus of care for people with dementia from a migration background?

**DOI:** 10.1186/s12889-020-08938-5

**Published:** 2020-05-26

**Authors:** Tim Schmachtenberg, Jessica Monsees, Wolfgang Hoffmann, Neeltje van den Berg, Ulrike Stentzel, Jochen René Thyrian

**Affiliations:** 1 German Center for Neurodegenerative Diseases (DZNE), Site Rostock/Greifswald, Ellernholzstraße 1-2, 17489 Greifswald, Germany; 2 Institute for Community Medicine, University Medicine Greifswald, Ellernholzstraße 1-2, 17489 Greifswald, Germany

**Keywords:** Dementia, Migration, Care, Needs, Healthcare services, Europe, National dementia plans, Dementia strategies

## Abstract

**Background:**

People with migration background and dementia are a vulnerable group. Providing care for this group is a public health challenge in Europe. An increasing number of countries are issuing national dementia plans, but a systematic overview of national dementia plans of European countries focusing on care for people with migration background is lacking. This study aims to illustrate how European countries identify the dementia-related needs of people with migration background and whether there are specific healthcare services for them at the national level.

**Methods:**

A qualitative analysis of national dementia plans of the EU and EFTA (European Free Trade Association) countries was carried out. Using the discourse analysis model according to Rainer Keller (2011), documents were systematically screened for their relation to migration via keyword and context analysis. The content of the migration-related sections was analyzed using the methods of paraphrasing, memos, comments, and open coding.

**Results:**

Twenty-three of the 35 EU and ETFA countries have a national dementia plan, ten of these documents refer to migration and one country (Austria) has a national dementia plan with a chapter on migration. Eight national dementia plans identify that people with migration background and dementia have special needs, and actions to care for this group are planned in nine countries. However, only Norway, Northern Ireland, and the Netherlands refer to available healthcare services for people with migration background. Overall, the topic of migration plays a subordinate role in the national dementia plans of European countries.

**Conclusions:**

The current lack of migrant-specific healthcare services in almost all European countries may lead to denying the right to appropriate care to a growing population. The topic of migration must be given greater attention in national dementia plans. European countries should develop strategies with specific services that address the needs of people with migration background. To improve comparability at the European level, a common definition of migration is needed. Further studies should include country-specific problems related to dementia and migration.

## Background

In 2010, the number of people with dementia (PwD) in Europe was estimated to be 9.95 million [1]. As a result of the increasing number of elderly people in most populations [2], the number of PwD is expected to rise by approximately 40% to 13.95 million in 2030 [1], imposing a major challenge for European societies. A subgroup of special vulnerability and importance is people with migration background (PwM) [3]. There are few data available which describe the number of PwM with dementia; for example, in Germany, the estimated number is 96,500 [4]. Furthermore, the prevalence of dementia within the migrant population will increase particularly strongly, since the number of older PwM is rising significantly [3]. In the EU, the number of PwM (born abroad) who are over 64 years of age has risen from 4.73 million in 2000 to 7.37 million in 2017 [5]. A study by Canevelli et al. estimates the number of PwM with dementia who are over 64 years of age in EU and EFTA (European Free Trade Association) countries to be almost 476,500 in 2017 [6]. Based on these figures, the proportion of PwM with dementia over 64 years of age among PwM from this age group is just under 6.5%. According to data from the “Dementia in Europe Yearbook 2019”, the proportion of PwD (with and without migration background) who are over 64 years of age among people from this age group in the EU and EFTA countries is almost 8.4% in 2018 [7]. This difference indicates the problem of a lack of diagnosis within migrant communities in many European countries [8]. One reason for the underdiagnosis of dementia in PwM could be a lack of adequate diagnostic tools [3]. Meta-analyses of dementia screening studies have shown that diagnose of dementia in migrants is more complicated and the diagnosis are less valid than in the majority population [8]. Furthermore, it has been hypothesized that PwM may develop dementia earlier than people without migration background [9, 10]. The increasing number of older PwM adds to the challenges for dementia care [11]. Various studies show that PwM use fewer dementia-related healthcare services [12–16]. Factors which may explain access issues for migrants include: convictions about dementia, lack of information and awareness about services, language barriers, stigmatization, and availability of services. Consequently, there is a risk that in the next years an increasing number of PwM will live with dementia and have no access to appropriate care [3]. There are efforts in different regions or countries to remedy these problems. For instance, in Germany, projects such as DeMigranz (Demenz Support Stuttgart) address specifically PwM with dementia and their relatives [17], or in the UK, the Somali dementia aware project (Somali Cultural Centre Camden London) focusses on PwD and caregivers from minority ethnic groups [18]. In encountering the challenges of dementia in their countries there is an increasing number of European countries issuing national dementia plans (NDPs) [19]. However, a systematic comparison of NDPs and their focus on care for PwM across Europe is missing.

This study aims to determine to what extent the special needs of PwM with dementia are identified in European countries, whether specific actions are taken at the national level to ensure their care and how attention is paid to the relationship between dementia and migration. The issue of actions for PwM with dementia is divided into two sub-questions: 1. Do specific healthcare services for PwM with dementia currently exist at the national level? 2. Are specific actions for the care of PwM with dementia planned? In this study, healthcare services at the national level are defined as all services involving healthcare, such as information, support, advice, diagnose, or treatment plans, which are not limited to specific regions, companies, or institutions and are referred to in official national documents by country representatives (e.g., representatives of health ministries, other members of government or representatives of national professional societies).

## Methods

For this study, a qualitative discourse analysis by Reiner Keller (2011) was conducted. This approach is based on the open research logic of qualitative social research. The proposed methods offer assistance in structuring the analysis process but do not represent regulations for the research process. The discourse analysis focused on the analysis of natural communication processes in different contexts. In the case of this study, discursive practices in the form of national documents were used. This knowledge-sociological approach aims to identify the processes and practices of knowledge production at the level of institutional fields. This method can be used to reconstruct whether and to what extent discourses establish or organize relations between phenomena [20]. Thus, this model is a suitable approach for revealing to what extent attention is paid at the national level to the relation between dementia and migration and what knowledge is available or imparted about PwM with dementia. With this method, an overview can be given of the institutionally stabilized knowledge resources regarding the care situation of PwM with dementia.

### Data sources

The information sources for the identification of NDPs were: the online platform of Alzheimer Europe [19], the Dementia in Europe Yearbook 2018 [21], and the Alzheimer’s Disease International’s overview of dementia plans from 2018 [22]. The online platform of Alzheimer Europe and the search engines Google and Google Scholar served as a database for the documents. These data sources were selected because they best meet the criterion of wide public accessibility. They should serve as a central source of information on dementia for relatives of people with dementia, care providers, and policymakers. This study includes the most recently published editions of the NDPs of the EU (20 NDPs from 31 countries) and EFTA countries (three NDPs from four countries). The range of the publication dates was 2009 to 2018. As England and Northern Ireland had no NDPs valid at the time of the survey, 1 June 2019, a supplementary national document valid in 2019 was examined for each of these countries. Furthermore, in Belgium, the dementia plan of the northern region Flanders was taken into account, as Belgium is a federal state, and dementia is only treated at the level of the Flemish (official language: Dutch) or French-speaking community (Wallonia, parts of Brussels) [23]. Consequently, there is no dementia plan for the whole of Belgium. The other two regions, Brussels-Capital and Wallonia, do not have a dementia plan. The data body comprises 25 documents.

### Procedures

These documents were systematically screened for their relevance to migration. The first step was to examine whether the documents included separate chapters on this topic. Then, the NDPs were screened for these key terms: minorities, minority, migration, culture, ethnic, background, migrant, sensitive, cultural, diverse, diversity, and language. If the migration topic was considered, a content analysis of the section in which it could be located was carried out. For this purpose, the contents were paraphrased, memos, and comments were added and the text passages were coded, using the strategy of open coding. The categories were derived from the contents of the documents. First, the content was roughly structured according to the categories’ problem description and actions and then fine-tuned according to the categories presented in Table 1. These categories were selected because they describe the content of the sections related to migration in the best way and include the central elements of the research question. Then, the contents of the statements were reconstructed in an interpretative-analytical way. Afterward, the results were interpreted and assessed [20]. The data were first interpreted individually for each country, then short country profiles were produced, and, in the end, the findings were compared.

**Table 1:** Reference of the national dementia plans of the EU/EFTA countries to migration

Countries	Dementia plans and migration reference			Sub-themes related to migration					Migrant-related needs and services		
	Dementia plan available	Reference to migration	Chapter on migration	Prevalence	Needs	Dementia diagnosis	Care	Utilization of formal services	Identification of special needs	Specific services available	Specific actions planned
Austria	Х	Х	Х	Х	Х	Х	Х	Х	Х	–	Х
Netherlands	Х	Х	–	Х	Х	Х	Х	–	Х	Х	Х
Norway	Х	Х	–	–	Х	Х	Х	Х	Х	Х	Х
Wales	Х	Х	–	–	Х	Х	Х	Х	Х	–	Х
Belgium /Flanders	Х	Х	–	Х	Х	–	Х	–	Х	–	Х
Northern Ireland	Х	Х	–	–	Х	–	Х	–	Х	Х	Х
England	Х	Х	–	Х	Х	–	–	–	Х	–	Х
Switzerland	Х	Х	–	–	Х	Х	–	–	Х	–	Х
Scotland	Х	Х	–	–	–	Х	Х	–	–	–	Х
Cyprus	Х	Х	–	–	–	–	Х	–	–	–	–
Czech Republic	Х	–	–	–	–	–	–	–	–	–	–
Denmark	Х	–	–	–	–	–	–	–	–	–	–
Finland	Х	–	–	–	–	–	–	–	–	–	–
France	Х	–	–	–	–	–	–	–	–	–	–
Greece	Х	–	–	–	–	–	–	–	–	–	–
Ireland	Х	–	–	–	–	–	–	–	–	–	–
Italy	Х	–	–	–	–	–	–	–	–	–	–
Liechtenstein	Х	–	–	–	–	–	–	–	–	–	–
Luxembourg	Х	–	–	–	–	–	–	–	–	–	–
Malta	Х	–	–	–	–	–	–	–	–	–	–
Portugal	Х	–	–	–	–	–	–	–	–	–	–
Slovenia	Х	–	–	–	–	–	–	–	–	–	–
Spain	Х	–	–	–	–	–	–	–	–	–	–
Bulgaria	–	–	–	–	–	–	–	–	–	–	–
Croatia	–	–	–	–	–	–	–	–	–	–	–
Estonia	–	–	–	–	–	–	–	–	–	–	–
Germany	–	–	–	–	–	–	–	–	–	–	–
Hungary	–	–	–	–	–	–	–	–	–	–	–
Iceland	–	–	–	–	–	–	–	–	–	–	–
Latvia	–	–	–	–	–	–	–	–	–	–	–
Lithuania	–	–	–	–	–	–	–	–	–	–	–
Poland	–	–	–	–	–	–	–	–	–	–	–
Romania	–	–	–	–	–	–	–	–	–	–	–
Slovakia	–	–	–	–	–	–	–	–	–	–	–
Sweden	–	–	–	–	–	–	–	–	–	–	–

### Language of national dementia plans

In the search for NDPs, primarily English and German terms were used. If no documents could be found in this way, a search was conducted using terms translated into the respective national language. 16 of the 25 NDPs examined were available in English and one NDP (Liechtenstein) in German, the native language of the first author. 5 of the remaining 8 NDPs (France, Luxembourg, Netherlands, Portugal, Spain) were translated using the translation program DeepL. The documents of Slovenia, Czech Republic, and Cyprus were screened after a Google search and with the help of the Google translator via keywords in the respective national language. The results of this study were discussed with various experts on the topic of dementia and migration from different EU and EFTA countries at a workshop in The Hague (Netherlands) in 2019.

## Results

**Fig. 1:**
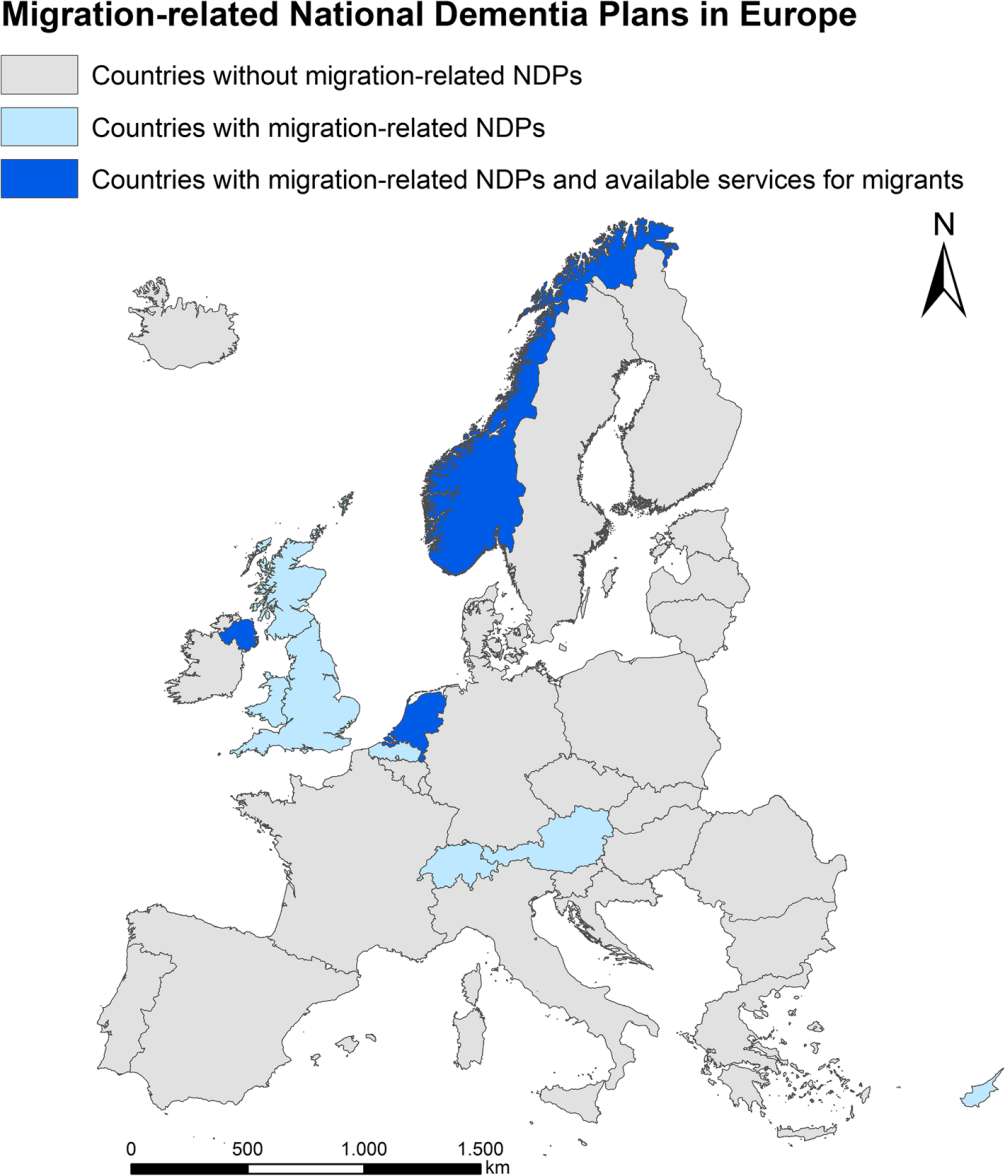
EU/EFTA Countries with migration-related National Dementia Plans and available healthcare services (source of the map in Figure 1: The map was created by the authors with the software ESRI®ArcGIS™ 10.5.1 Esri Inc., Redlands/California (USA), for the use of which a license was required. Geo data source: European Commission, Eurostat (ESTAT), GISCO)

Twenty-three of the 35 EU and EFTA countries have issued a national dementia plan (NDP). More than half (13) of the countries with a NDP do not refer to migration. Ten countries discuss this topic in their documents (Austria, Switzerland, the Netherlands, Belgium/Flanders, England, Scotland, Wales, Northern Ireland, Norway, and Cyprus). However, only one state (Austria) has a NDP with a chapter on migration (Table 1). The NDPs with migration reference differ considerably in terms of the scope of the reference, the range of topics, and the focus and depth of the content. The Austrian Dementia Report, for example, devotes four full pages in detail to PwM with dementia. Other NDPs, such as those in Scotland, Switzerland, or Cyprus, which minimally refer to this issue, dedicating only a few sentences (Scotland: early diagnosis and care, Switzerland: migrant needs and diagnostic challenges, Cyprus: dementia risk and care). Eight of the ten NDPs with migration reference identify that PwM have special needs in dementia care. Nine countries are planning migrant-related actions. However, only Norway, Northern Ireland, and the Netherlands point to currently available specific healthcare services for PwM at the national level (Fig. 1).

### Overview of country-specific-strategies

#### Austria

The Dementia Report 2014 identifies the problems of a later diagnosis of dementia and the lower utilization of care services, especially by Turkish migrants. Language barriers, cultural factors, standardized diagnostic tests, which are inappropriate for PwM, and the considerable lack of migrant-specific care, especially with regard to dementia prevention, are mentioned as reasons for this. The main problem is insufficient networking between dementia specialists and migration experts. To better address the needs of PwM, native speakers with intercultural experience should be employed, caregivers trained, and staff in migrant counseling centers made aware of available services [24].

#### Belgium/Flanders

The dementia plan for Flanders (2016–2019) notes that the prevalence of dementia among migrants is significantly higher than among people without migration background and that there is a relation between ethnicity and the development of dementia. Flanders has set the goal of developing culturally sensitive care services. For this purpose, Flanders would like to use the knowledge on culturally sensitive care for migrants from Erasmushogeschool’s research and implement it in specific projects such as dementia-skilled training and the consultation platform of dementia. Thus, the Dementia Consultation Platform (Overleg Platform Dementie) can initiate targeted neighborhood initiatives and bring together care stakeholders and relevant local stakeholders [25].

#### Cyprus

The 2012–2017 dementia plan identifies ethnicity as a risk factor for dementia. Cyprus aims to ensure equal access to diagnostic tests, treatments, medicines, and care for all population groups and to prevent discrimination in dementia care based on race or origin. A strategy to implement this aim is not mentioned [26].

#### England

The Dementia Strategy 2009 and the All Party Parliamentary Group Report 2014 do not identify ethnic minorities as a vulnerable group for dementia. However, it is recognized that they may have special needs. England would like to provide them with specific information. Curricula for dementia-specific training of health and care professionals should address the needs of ethnic minorities [27]. In addition, ‘dementia leads’, people with a particular responsibility to ensure quality care for people with dementia that acting as a linkage between local organizations and service providers, should ensure that these needs are considered [28].

#### Netherlands

While the public version of the dementia standard 2016 does not address migration [29] the version for professional service providers refers to the fact that dementia is increasingly common among people of non-Dutch origin and that this group has special needs in dementia diagnosis and care. The Netherlands pays particular attention to migrants in early detection and prevention. PwD and their relatives are offered activities that are oriented to their cultural background. In the future, special attention will be given to migrants with dementia in the provision of housing [30].

#### Northern Ireland

While the Dementia Strategy 2011 does not refer to migration [31], the Northern Ireland Learning and Development Framework for Dementia 2016 lists some actions to address the special needs of migrants in dementia care. Service providers should be sensitized to identify cultural differences and their effects on PwD and trained in specific communication skills. Northern Ireland has a self-assessment tool for service providers that has a special focus on people with dementia from different cultural backgrounds [32].

#### Norway

The Dementia Plan 2020 describes the problems that older migrants often receive healthcare services only at an advanced stage of the disease and that language barriers between professionals and patients pose a threat to patient safety. It refers to a plan to take the needs of Sami and language minorities into account in the construction of nursing and residential homes. Norway would also like to involve PwD from different cultural groups in the development of a pilot project for post-diagnostic observation. Since 2015 there is a national program for PwD from Sami and language minority groups [33].

#### Scotland

According to the Dementia Strategy 2017–2020, Scotland wants to ensure that cultural aspects are taken into account in early detection and that competent local services and post-diagnostic support channels are available for people from protected characteristic groups with dementia. However, it is not mentioned which specific groups these are. Further research is also announced to increase understanding and awareness of dementia among different population groups [34].

#### Switzerland

The Dementia Strategy 2014–2019 describes the growing proportion of the migrant population in older age groups and the resulting changes in quality requirements of healthcare services. It also addresses the problem of language barriers associated with the diagnosis of dementia and the failure of common diagnostic tests in migrants. Switzerland intends to extend the current program on migration and health to include dementia [11].

#### Wales

The Dementia Plan 2018–2022 describes the problems of diagnosis, which are complicated by cultural and language interpretations, the lower utilization of care services, and the change in communication skills during the dementia process in ethnic minorities. Wales wants to ensure that these groups have easy access to suitable services and provide culturally appropriate care and support. To this end, linguistically diverse and culturally appropriate diagnostic tools are to be used, staff training courses conducted, specific information materials developed and the individual culture of PwD must be evaluated [35].

### Comparisons between countries

In most NDPs the focus is on the problem description. The most frequently addressed problems are: difficulty or later diagnosis and lower utilization of care services. Cultural and language barriers, as well as inappropriate diagnostic instruments, were named as causes for this. While in some countries such as the Netherlands or Belgium/Flanders, PwM are identified as a risk group for dementia, and in almost all countries as a risk group for underdiagnosis and a lower level of care, England does not perceive PwM as a vulnerable group. Concerning the planned actions, attention will be paid to the conception of specific information materials, training of caregivers, medical and nursing professionals or staff of migrant counseling centers, and the development of language and culturally appropriate diagnostic tools. Additionally, several countries would like to take greater account of cultural aspects in the prevention or early detection and the needs of PwM regarding living space.

### Level of migration in EU and EFTA countries and relationship with migration reference of national dementia plans

In the individual countries, the importance of the topic migration varies due to the different extent of immigration and emigration, the different size of the migrant population, and the different history of migration. On the one hand, there are immigration countries such as Austria, Belgium, France, Germany, Italy, Spain, Sweden, Switzerland, and England, whose migrant population (born abroad) consists of more than one million people (Germany: 13.1 million, as of 2019), where the proportion of migrants in the total population is more than 10 % (Switzerland: 29.9%) and where the net migration figure per year (immigrants minus emigrants in the last 5 years before 2020) is more than 100,000 (Germany: 2.7 million). On the other hand, there are some countries such as Bulgaria, Romania, Slovakia, the Czech Republic, and Hungary, whose migrant population has also grown over the past two decades but currently still accounts for a small percentage of the total population (5% or less, Romania: 2.4%) and countries such as the Baltic States and Poland, whose migrant population is shrinking and some of which have a clearly negative net migration (Poland and Lithuania) [36]. Putting these results in relation to the migration relevance of the individual NDPs does not provide a clear picture. There are immigration countries such as Austria and Belgium/Flanders that refer in detail to the topic of migration in their NDPs, while other immigration countries such as France, Italy, or Spain do not consider this topic. The average migrant proportion in the total population is not higher for countries with migration-related NDPSs (18.09%) than for countries whose NDPs do not take this topic into account (18.75%) (own calculations based on figures from the International Organization for Migration (IOM)). However, it is striking that no country whose migrant proportion of the total population is below 10%, whose migrant population is shrinking or whose net migration is negative has an NDP with a migration reference. In addition, the net migration rate (per 1000 inhabitants) is significantly lower in countries without migration-related NDPs (2.78) than in countries whose NDPS considers this topic (4.57) (data basis: IOM) [36]. Two conclusions can be drawn from this analysis: 1. A high proportion of migrants and the status of an immigration country does not automatically mean that the topic of migration is taken into account in a country’s NDPs. 2. A low proportion of migrants and the status of an emigration country seems to mean that the topic of migration is not taken into account in a country’s NDP.

## Discussion

This study aimed to determine to what extent attention is paid to the relationship between dementia and migration in the EU and EFTA countries, whether special needs of PwM with dementia are identified and if specific actions for the care of PwM with dementia are taken at the national level. A central finding of this analysis is that the topic of migration plays a subordinate role in the NDPs of European states. In more than half of the NDPs, the topic of migration is not considered. In most of the NDPs reviewed, the topic of migration is only briefly acknowledged and addressed. The Austrian Dementia Report is the only document containing a separate chapter on migration issues for PwD. Eight of ten countries identify that PwM have special needs that are relevant to dementia care (different communication, language, religious, spiritual, and cultural needs, different decision-making, preferences/expectations related to disease, diagnosis, and treatment). Nine countries are planning actions for dementia care of PwM. A study on addressing the issue of dementia among PwM in NDPs of the member states of the World Health Organization found that nine of 32 NDPs mentioned migration and eight of them, including five European countries, proposed actions for migrants or ethnic minorities [37]. This analysis indicates that in 32 of 35 European countries, there are large national policy gaps regarding care services for PwM with dementia. Only the Netherlands, Northern Ireland, and Norway refer to existing services for this target group.

In the Netherlands, special attention has been paid to PwM in the early detection and prevention of dementia. Overall, the Netherlands adopts an inclusive approach regarding healthcare for PwM. Healthcare services for this vulnerable group are organized through municipal service providers [38, 39]. However, the Dutch government, together with a number of organizations such as Pharos (Knowledge and Advice Centre for Migrants and Health), the Health of Immigrants Netherlands Foundation (SGAN), or the Dutch Association of General Practitioners (NHG), is trying to support local healthcare providers, improve migrants’ access to healthcare services, and establish nationwide advisory and information structures [40–42].

In Northern Ireland, the healthcare for PwM is organized through the Department of Health, Social Services and Public Safety (DHSSPS). Together with the Public Health Agency, the DHSSPS develops guidelines for screening PwM and provides resources for medical services. The Department promotes an inclusive healthcare system. Primary care is provided at the municipal level, where various organizations support PwM in meeting their healthcare needs [43]. Northern Ireland has developed a self-assessment tool for service providers that contains a whole questionnaire with items around the topic of migration.

Norway is improving the skills of staff members working with language minorities and developing a program of post-diagnostic follow-up models including PwD and their relatives from other cultural groups. At the municipal level, PwM are offered both mainstream primary healthcare services and specialized services (by the medical professional service). At the national level, Norway also has a competence center for migration and minority health (NAKMI). Together with further national organizations such as the National Competence Centre for Ageing and Health and the Ministry of Health, the NAKMI has developed nationwide available healthcare services for PwM with dementia [44].

Such concepts, communicated at the national level by representatives of the state, can help to raise awareness on the topic of migration among providers of dementia-specific care services and can serve as models of good practice for other countries.

The results of this study are based on the analysis of NDPs. As some EU and EFTA countries (12) do not have such documents, more countries may have specific national healthcare services for PwM with dementia. In addition, some NDPs have been published several years earlier than others. The topic of dementia and migration should be more important in large parts of Europe today than it was a few years ago due to the increase in prevalence and the growing number of older migrants. Thus, the different dates of publication can also be a cause for the country-specific differences concerning the reference to migration. A reason already discussed in the results section are the different levels of importance of migration in individual countries. This study shows that immigration countries with a large proportion of migrants are more likely to have migration-related NDPs than emigration countries with a low proportion of migrants. Another striking feature is the disparate terms used in the context of migration (e.g. language minorities in Norway [33], people of non-Dutch origin in the Netherlands [30] or people from different cultural backgrounds in Northern Ireland [32]) and the unequal definition of the term “migrant”. For example, in the UK a migrant can be a person “whose country of birth is different to their country of residence”, “whose nationality is different to their country of residence” or “who changes their country of usual residence for a period of at least a year” [45]. In Germany, a person is considered to have a migration background if she or he or at least one parent was not born with German citizenship [46]. The Alzheimer Europe report “The development of intercultural care and support for people with dementia from minority ethnic groups” discusses various concepts and terms frequently used in scientific articles and policy documents, such as “ethnic minority group”, “migrant”, or “immigrant”. In this report “ethnic minority groups” are defined as groups of people who share a common cultural identity and who differ in some way from the ethnic majority group in the respective country [3]. Ethnic, linguistic, or cultural minorities are numerically and in terms of ethnicity, language, or culture a minority [47]. The concepts of “migrant” and “immigrant” are described as unclear, as there is no generally accepted definition. These terms are often defined using criteria such as foreign birth, foreign citizenship, or temporary/permanent movement to a new country. Frequently “migrant” and “immigrant” are associated with ethnic or religious minorities, asylum seekers and refugees, or sometimes even used interchangeably, although these terms must be separated [3]. The term “migrant” includes all people who have crossed an international border [48] and are staying in the country that is new to them, while the concept “immigrant” refers exclusively to people who are or want to be resident in their new country [49] and are seeking a permanent settlement [50]. Heterogeneity in the definitions of migration across Europe is likely to impact the attributed importance of migration concerning dementia. For a more exact determination of the importance of the topic dementia and migration in Europe and better comparability of country-specific data (prevalences, healthcare services, dementia strategies), an uniform use and definition of terms such as “migrant” would be essential [3]. In this context, definitions based on the country of birth of the individual person (born abroad) or the last country of permanent residence should be used. The definition of the United Nations, which is used by many experts, is recommended: An “international migrant” is “any person who changes his or her country of usual residence” [51]. In spite of its limitations, this study provides an overview of the extent to which the topic of migration is taken into account in the NDPs of the European states and which thematic emphases are set if the topic is considered. The central knowledge gained from this study is that, on the one hand, it presents the migrant-specific dementia strategies of the EU and EFTA countries and points to gaps in care in the individual countries. On the other hand, the comparison of countries also shows similarities and differences between the NDPs of the European states. Thus, this analysis provides the first overview of country-specific strategies for the care of PwM at the European level.

## Conclusions

This study supplements the current literature with a systematic description of the role that migration plays in the NDPs of the EU and EFTA countries and the actions communicated by some to ensure the care of PwM. At present, the topic of migration in the context of dementia plays a subordinate role at the national level in most European countries, and there are hardly any specific care plans for PwM. Since a significant increase in the prevalence of dementia among PwM is expected in the coming years and this group is currently utilizing fewer healthcare services, partly due to inappropriate services, greater attention must be paid to the topic of migration in NDPs. All European countries should develop a strategy at the national level and define services that address the individual, language, and cultural needs of PwM. However, from our analysis specific recommendations cannot be given. There is a need for further research and discussion about the implementation of current governmental agendas. Furthermore, more attention should be drawn to other forms of support for this vulnerable group. However, we assume that the current lack of migrant-specific care at the national level, if not remedied in a timely manner, may lead to a growing population being excluded from care.

## Data Availability

The data analyzed in this study are publically available via the official website of Alzheimer Europe (https://www.alzheimer-europe.org/Policy-in-Practice2/National-Dementia-Strategies).

## Abbreviations

EFTA: European Free Trade Association
IOM: International Organization for Migration
NDP: National dementia plan
NDPs: National dementia plans
PwD: People with dementia
PwM: People with migration background
